# Revisiting percutaneous balloon mitral valvotomy technique and safety in various population: an evidence-based case report and literature review

**DOI:** 10.3389/fcvm.2024.1334444

**Published:** 2024-01-26

**Authors:** Faisal Habib, Brian Mendel, Rivhan Fauzan, Ali Nafiah Nasution

**Affiliations:** ^1^ Department of Cardiology and Vascular Medicine, Haji Adam Malik General Hospital, University of Sumatera Utara, Medan, Indonesia; ^2^ Department of Cardiology and Vascular Medicine, Sultan Sulaiman Government Hospital, Serdang Bedagai, Indonesia

**Keywords:** atrial fibrillation, mitral stenosis, over-the-wire, percutaneous balloon mitral valvotomy, pregnancy, rheumatic heart disease, veno-arterial loop

## Abstract

Percutaneous balloon mitral valvotomy (PBMV) is a good and preferred therapy choice over surgical commissurotomy for patients with rheumatic mitral stenosis (MS). However, interventional cardiologists must recognize that treating patients with rheumatic MS poses unique challenges for each patient, especially in special populations such as pregnant patients or patients with arrhythmias like atrial fibrillation (AF), which can complicate procedures. Based on information from observational studies, PBMV may be a safe and efficient treatment for improving outcomes in MS women who do not have substantial subvalve illness in a specific demographic. A successful PBMV helps to tolerate hemodynamic changes during pregnancy and dramatically reduces mortality. However, there is a paucity of studies on women with poor valve morphology who are not contraindicated, and it has to be seen if PBMV is used in these situations during pregnancy. Conversely, AF leads to a lower PBMV success rate as well as worse long-term and in-hospital outcomes.

## 1 Introduction

Patients with rheumatic heart disease (RHD) continue to have a high frequency of mitral stenosis (MS) in emerging nations (1–3). For patients with rheumatic mitral stenosis, percutaneous balloon mitral valvotomy (PBMV) is a suitable and preferred treatment option over surgical commissurotomy. PBMV has better results, a shorter hospital stay, and lower risk than surgical valvotomy. In recent decades, PBMV techniques and safety have also improved (4–6).

Interventional cardiologists must understand, though, that managing patients with rheumatic MS presents different challenges for each patient, particularly in special populations like pregnant women or patients with electrophysiological disorders like atrial fibrillation (AF), which can make procedures more difficult (7, 8). Treatment for severe mitral valvular stenosis globally often consists of percutaneous balloon mitral valvotomy with an Inoue balloon. Interventional cardiologists occasionally encounter difficulties crossing the mitral valve (MV) for a variety of reasons that result in the procedure failing. One of the relevant causes of procedural failure is the incapacity to cross the mitral valve (9).

Therefore, we would like to present an evidence-based case report in this paper, which will be followed by a review of the literature on the various PBMV techniques available in patients with MS RHD, a comparison of PBMV techniques in other populations, such as atrial fibrillation, left atrial thrombus, and pregnant MS RHD women, and a comparison of the clinical outcomes.

## 2 Case description

A 23-year-old woman presented to our center with shortness of breath with minimal effort (New York Heart Association functional class/NYHA fc III-IV). Her physical examination showed pulsus deficit, an accentuated first heart sound, and a rumbling murmur over the cardiac apex. Echocardiography confirmed the diagnosis of severe rheumatic mitral stenosis with a mitral valve area of 0.4 cm^2^ and a mean pressure gradient of 12 mm Hg across the valve. With a Wilkin's score of 5/16 and after the exclusion of left atrial appendage thrombus by transesophageal echocardiography, the patient was considered for percutaneous transvenous mitral commissurotomy.

**Figure 1:**
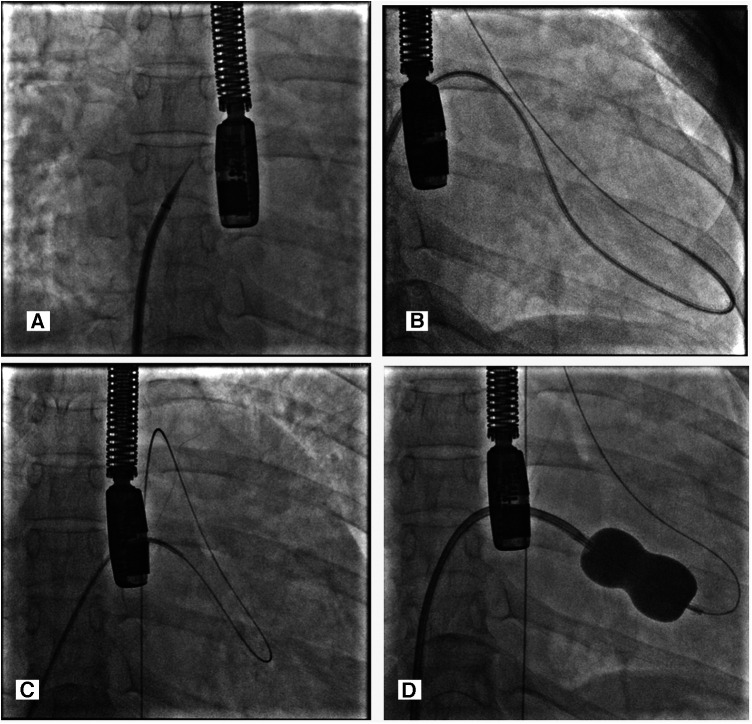
(**A**) Transeptal puncture with guiding TEE. (**B**) MP-6fr catheter via Mullins longsheath introducer into the left ventricle, inserting an exchange-length 0.035” Terumo wire into the left ventricle and across the aortic valve into the descending aorta. (**C**) Veno-arterial loop—0.035” exchange length Terumo wire directed from the LV to the descending aorta with the help of a 6Fr MP catheter inside Mullin's longsheath and it being snared with a snare kit inside a JR diagnostic catheter. (**D**) The Inoue balloon was dilated over snared Terumo wire to dilate the commissural part of the mitral valve.

Prior to the procedure, the patient was intubated, and transesophageal echocardiography was performed to guide the transseptal puncture and evaluate the severity of the mitral stenosis with a planimetry mitral valve annulus (MVA) of 0.4 cm^2^ and a mean gradient of 11 mmHg. The right femoral artery and vein were accessed using the 7Fr and 8Fr access sheaths. Standard steps were followed using a Brockenbrough needle through 8Fr Mullins introducer sheath for an interatrial septum (IAS) puncture (Figure 1A), and the left atrium was entered. Then a 0.025” spring guide wire (LA wire) was introduced into the LA. The 8Fr Mullins introducer sheath was exchanged with the 14Fr septal dilator. The IAS was dilated with the 14Fr septal dilator. Following this, a 26 mm-size Inoue balloon catheter was introduced into the LA over a 0.025” spring guide wire. Several attempts to negotiate the Inoue balloon into the LV across the MV but failed to cross the MV. We withdrew the Inoue balloon and took the multipurpose-6Fr catheter via Mullins longsheath introducer into the left ventricle, inserting an exchange-length 0.035” Terumo wire into the left ventricle and across the aortic valve into the descending aorta. However, the wire was not supporting the Inoue balloon's entry into the left ventricle (Figure 1B). The Terumo wire was snared in the descending aorta with a snare kit supported by a JR-6Fr diagnostic catheter. We could then advance the Inoue balloon system into the left ventricle (LV) across the veno-arterial rail, which provided more support and directed it toward the apex of the left ventricle (Figure 1C). PBMV was then completed with serial dilatations of Inoue balloon no. 26 mm (Figure 1D).

**Figure 2:**
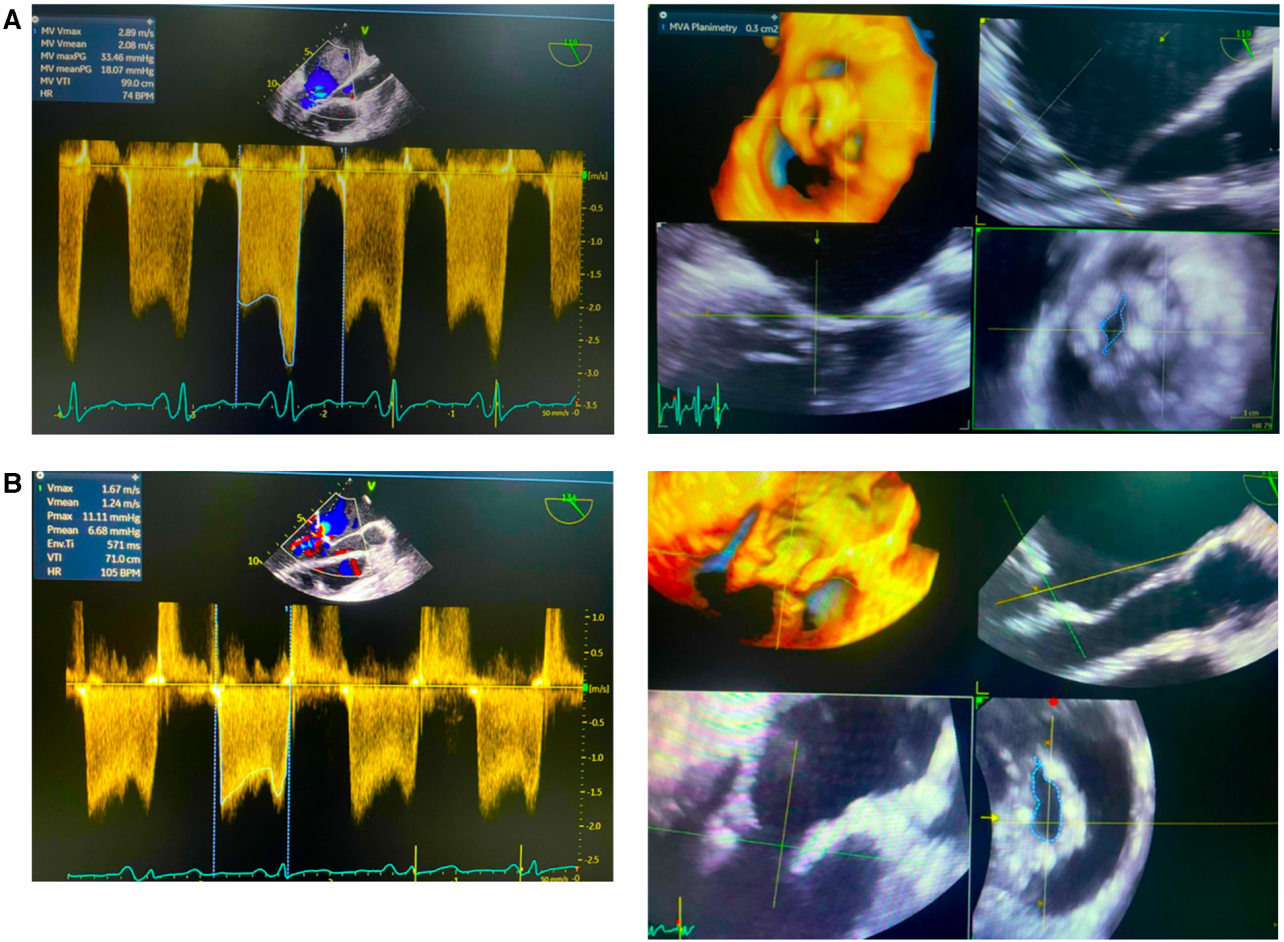
Echocardiography pre- and post- percutaneous balloon mitral valvotomy (PBMV). (**A**) Initial data showed mean valve area (MVA) plannimetry was 0.3 cm and mean valve gradient (MVG) 18.7 mmHg. (**B**) Post PBMV showed mean valve area (MVA) plannimetry was 1.0 cm and mean valve gradient (MVG) 6.68 mmHg.

The procedure was successfully completed, as the MV area increased to 1 cm^2^, and the mean mitral valve gradient was reduced from 11 to 5 mmHg with no mitral regurgitation or hemodynamic disturbances observed (Figure 2). No complications occurred during or after the procedure. The patient was discharged in a stable state, and on follow-up, she has improved exercise tolerance.

## 3 Discussions

### 3.1 Percutaneous balloon mitral valvotomy technique in rheumatic heart disease patient

PBMV is a standard procedure employed for the treatment of symptomatic rheumatic MS across all age groups with a favorable MV anatomy (4–6). In forty years, not much has changed in terms of the instruments employed; nonetheless, increased procedural safety has been made possible by advances in imaging technology and the field of interventional cardiology. Throughout the course of the last 40 years, PBMV procedures have evolved, bringing about advances in preprocedure evaluation, vascular access, intraprocedural imaging guidance, anticoagulation and anesthesia. (see Table 1) (10). In most cases, an Inoue balloon with a preshaped stylet is used to cross the MV. Anatomical distortion of the interatrial septum with a huge LA sometimes leads to a low or anterior septal puncture. Sometimes, after entering the LA, entry into the LV is cumbersome due to the abnormal position of LV inflow, critical MS, and severe subvalvular disease (9, 11). Degenerative and rheumatic valves differ anatomically and pathologically in a number of ways, including the percutaneous valve landing zone. When contemplating percutaneous intervention, these have inadequate documentation and can call for specialized treatments (12).

**Table 1:** The development of percutaneous balloon mitral valvuloplasty (PBMV) procedures (10).

1. Preprocedure assessment: using commissural anatomy and 3D echo to size balloons and assess appropriateness is becoming more common.
2. Several updated scoring schemes; Wilkins–Weyman echo score stays unchanged
3. Access: In keeping with the general trend toward large vascular access in treatments for structural heart disease, many locations now employ ultrasound guidance.
4. Use of a sheath: although deemed undesirable due to possible damage to the Inoue balloon, this is now seen as flawless; the standard sheath is 14F, which makes deflating the balloon rather challenging, or 16F.
5. Anticoagulation was formerly limited to heparin administered following transseptal puncture; however, this has evolved to include full anticoagulation following successful transseptal puncture and partial anticoagulation following venous access. Certain locations regularly perform PBMV while maintaining full anticoagulation throughout.
6. Transitioned from using only fluoroscopy (which is still used in some underdeveloped nations) to intracardiac echo (ICE) or transesophageal echo (TEE) guiding during transseptal puncture. Most operators prefer mid-mid to inferior-posterior puncture sites because to their increased ability to precisely find these areas in the fossa ovalis.
7. Although enhanced ICE technologies and hybrid computed tomography, cath, and noninvasive imaging are also used, TEE continues to be the principal imaging guidance overall.
8. In most cases, patients receiving TEE guidance are placed under general anesthesia as opposed to deep sedation.
9. Balloon sizing: primarily still based on the height formula, but increasingly including intracommissural measurements and 3D imaging
10. Vascular closure: “preclose” technique or Perclose employing a figure of eight skin suture

Turi ZG. The 40th anniversary of percutaneous balloon valvuloplasty for mitral stenosis: current status. Structural Heart. 2022; 6(2022):100087.

.

**Figure 3:**
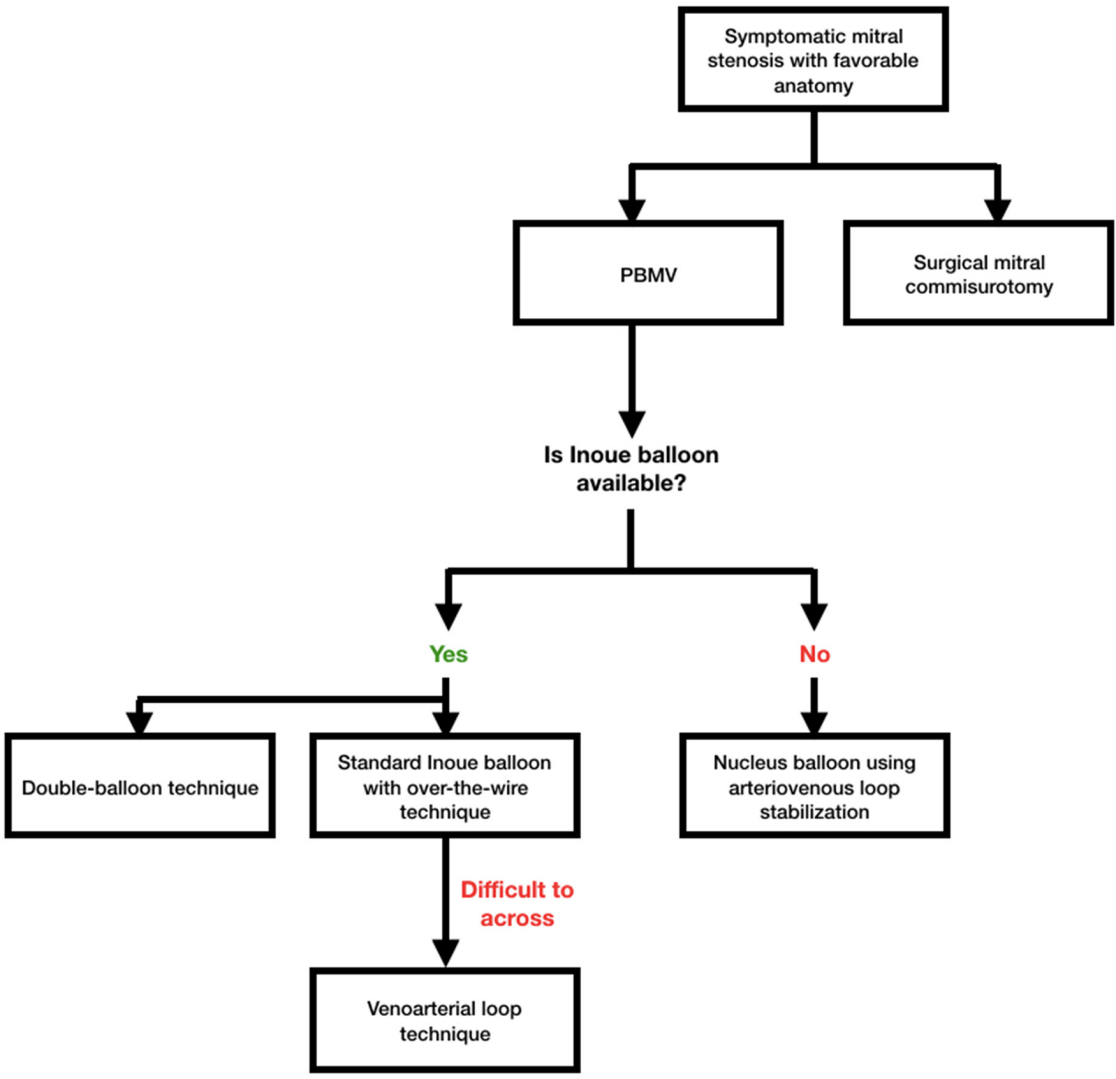
Proposed algorithm for percutaneous baloon mitral valvotomy (PBMV) in symptomatic mitral stenosis patients with favorable anatomy. Standard Inoue balloon with over-the-wire technique should be the default option in settings where Inoue balloon is offered. The venoarterial loop approach could be employed for stabilization if the wire is challenging to get through. Nucleus balloon employing arteriovenous loop stabilization may become an option when Inoue balloon is not a viable choice. The usual single Inoue balloon technique is preferable because the double-balloon procedure had a higher rate of complications with comparable outcomes.

Many authors have described their experiences with different techniques such as the reverse loop technique where the LV is entered by forming a guide wire loop in the LA, which allowed the balloon catheter to cross the MV, the double loop technique whereby with anticlockwise rotation of the J-shaped stylet a double loop is made in the LA, making the Inoue balloon face mitral inflow to enter the LV, and the vertical approach technique using LV pressure as a guide for LV entry. Although the double-balloon procedure proves effective, it is more technically difficult and takes longer to complete, which could result in unintended issues. In actuality, PBMV utilizing a single Inoue balloon produces effectiveness that is equivalent to that of the double-balloon procedure while posing a lower procedural risk (9–14). Figure 3 depicts the PBMV method we suggested.

Vasu et al. (9) used a 0.035”, 260 cm long hydrophilic guide wire through the Accura balloon to cross the mitral valve; the same wire was advanced to the aorta across the aortic valve into the right subclavian artery and right ulnar artery, then anchored the wire by flexing the right elbow to support and track the Accura balloon over the wire into the LV. Nanjappa et al. (15) mentioned to cross the MV, they removed the Inoue balloon from the LA and crossed the MV and aortic valve using 0.035” wire through Mullins sheath and multipurpose catheter, then snaring the wire in the descending aorta to form a venoarterial loop and advance the Accura balloon system into the LV. However, the potential problem with this technique is that all the manipulations need to be gentle because an arteriovenous loop can cut through the valve and aorta unless it is protected by a catheter, especially when there is a critical mitral stenosis situation needing sufficient pull and push to prevent cardiac perforation, ventricular arrhythmias, a tear of the mitral valve, and stroke. In our case, a 0.035”-long Terumo wire was also directed to the aorta, crossing the mitral valve and LV using a multipurpose catheter. Then, the terumo wire was forwarded into the descendent aorta, snared in the descending aorta from the right femoral artery sheath, and pulled out from the sheath. The venoarterial loop was formed with good support by the JR diagnostic catheter. This technique gives more support for Inoue balloon entry across LV compared to over the wire technique.

Thakur et al. (11) reported successful PBMV with a rapid snare sliding technique similar to the venoarterial loop, where this technique was simple and less complicated with the least risk as 0.025” hydrophilic wire will not cause major trauma to the heart chambers and is easily threaded through the Inoue balloon. The time and manipulations required to snare and make the Inoue balloon catheter cross MV are less than attempting various maneuvers to cross MV, decreasing the procedure time, complications, and radiation exposure. It would be a useful, safe, and less complicated alternative in patients with difficult LV entry during PBMV (16, 17). The Stefanadis technique uses a specially made steerable catheter to enter the left atrium retrogradely via the left ventricle in other difficult-to-enter LV instances. This avoids the need for a transseptal puncture, which is a necessary step in other balloon mitral valvuloplasty techniques (18).

In nations with little financial resources, the expense of the PBMV process continues to be a barrier, which results in the frequent reuse of disposable catheters. A metallic valvotomy device that is reusable has been created in order to get over this restriction. The apparatus is comprised of a metallic cylinder that may be detached and has two articulated bars attached to its distal end. The proximal end of the disposable catheter is linked to an activating pliers. Ninety-two percent of the 153 patients in the clinical experience had good outcomes, including an increase in the mitral valve. The device's results are encouraging and on par with the current balloon approach, at the very least (19).

### 3.2 Percutaneous balloon mitral valvotomy in patient with atrial fibrillation

Rheumatic heart disease (RHD) is routinely disregarded by politicians, the media, and even the medical community, despite being a leading cause of morbidity and mortality among the youth in many parts of the world. The current guidelines for managing patients with rheumatic atrial fibrillation (AF), who are frequently younger, female, and have less comorbidities, are not applicable to those with nonvalvular AF (8). The global prevalence of AF in RHD was found to be 32.8% in a meta-analysis of 83 studies from 42 countries, with significant heterogeneity (4.3%–79.9%) depending on the level of development of the nation. As people age, the prevalence of AF in RHD rises, reaching 39.7% in adults compared to 7.6% in children and adolescents (20). The management of rheumatic AF is contingent upon the necessity of valve intervention in addition to stroke prevention. When deciding whether to use a rate or rhythm control method, valve intervention is not necessary. In the event that valve intervention is required, the choice of rhythm or rate control technique is made at that moment (8).

According to Aslanabadi et al. (21), compared to patients with sinus rhythm (SR), patients with AF who received PBMV had far lower immediate success rates, a higher death rate, and greater rates of short- and long-term complications. Furthermore, compared to patients with SR, MS patients with AF prior to PBMV were considerably older, and they also had higher mitral echocardiographic Wilkins scores, NYHA-FC, larger MR, and mTMPGs. According to Leon et al. (22), AF patients exhibited higher mean LAP following PBMV and smaller MVA and TMPG prior to PBMV. Patients with AF had greater re-stenosis and a smaller MVA following PBMV, according to research by Fawzy et al. (23).

The correlation between AF and advanced age, higher NYHA-FC, and elevated mitral valve with more severe morphological and structural alterations, AF is a presentation of long-term rheumatic MS that may predict a less favorable response to treatment and a higher frequency of adverse events, according to the Wilkins score. The success rates of PBMV and SR patients in the Maatouk et al. (24) trial were statistically comparable (89.7% and 92.3%, respectively). According to Liu et al. (6), there is evidence to suggest that patients with AF may benefit from PBMV less than those with SR.

### 3.3 Percutaneous balloon mitral valvotomy in patient with left atrial thrombus

About 3%–13% of mitral stenosis (MS) patients experience left atrial (LA) thrombus, which is typically regarded as a contraindication to PBMV. A potentially fatal consequence, systemic embolization affects 0.3%–0.8% of patients during or soon after the procedure. The majority of the time, this complication is generally assumed to be caused by the dislodgement of thrombus that was already there prior to the treatment; but, in certain instances, embolization of catheter-induced thrombi or calcific embolisms may be to blame (25).

Based on their position, extension, and motility, LA thrombus were categorized as Type Ia (LA appendage clot confined to appendage), Type Ib (LA appendage clot protruding into LA cavity), Type IIa (LA roof clot limited to a plane above the plane of fossa ovalis), Type IIb (LA roof clot extending below the plane of fossa ovalis), Type III (layered clot over the interatrial septum), Type IV (mobile clot which is attached to LA free wall, roof, or IAS), and Type V (ball valve thrombus). A total of 108 patients with type Ia, type IIa, and Ib LA thrombus who met the inclusion criteria received PBMV using the over-the-wire approach in a research conducted by Manjunath et al.. Following the procedure, the pulmonary artery systolic pressure, LA mean, mitral valve area, and mitral valve gradient all showed a significant and comparable improvement. A transient ischemia attack did, however, happen in one instance six hours following a successful PBMV. When carried out by skilled operators, systemic thromboembolism, technical difficulties, and other issues are quite uncommon (25).

### 3.4 Percutaneous balloon mitral valvotomy during pregnancy

The most prevalent valvular heart condition that complicates pregnancy is still mitral stenosis (MS), which increases the risk of maternal death in cases of severe impairment, particularly in low- and middle-income nations. After a successful procedure, the hemodynamic tolerance to pregnancy and labor-related alterations improves; however, there is still a danger of potentially fatal acute mitral regurgitation (MR) and worries about radiation exposure to the fetus (26–29). In their systematic review, Sreerama et al. (5) revealed that 21 observational studies with a total of 745 pregnancies were considered. In 93.6 percent of cases, PBMV was successful in improving both the valve area and the patient's symptoms. Compared to patients who had a successful treatment, maternal mortality was greater in those who had an unsuccessful PBMV.

Pregnant MS patients are at risk for hemodynamic alterations during pregnancy, particularly if they fall into NYHA class 3 or 4. This puts their chance of dying during childbirth at 6%–10%. By opening the mitral valve, PBMV lowers the left atrial pressure and transmitral gradient, which lowers the risk of pulmonary edema. By increasing area and decreasing pressure gradients, PBMV performed in the second trimester—well ahead of the peak hemodynamic alterations in pregnancy, which occurs around 32 weeks—may be beneficial. In contrast to the increased mortality rates (6%–10%) documented for untreated MS pregnant women, the combined estimates of 0.01% (0.0–0.76) imply enhanced resilience to hemodynamic alterations after PBMV, hence enhancing maternal outcomes (5, 26–29).

While most published research employed the Inoue technique, which reduces fluoroscopic time, only a small number of studies reported using the over-the-wire technique to use the Joseph mitral valvuloplasty balloon catheter (JOMIVA). There have been reports of similar success rates and little radiation exposures with other devices, like the Accura single lumen balloon catheter (30).

Two cases reported miscarriage, probably as a result of the procedures; two cases reported stillbirth; and two cases reported stillbirth after a failed BMV. Hypoxia or hypotension brought on by uncontrollable heart failure may be the cause of this. The investigations’ obstetric outcomes include cesarean delivery, stillbirth, premature birth, and fetal growth limitation. Rather than the procedure itself, this could have to do with how serious the sickness is. Malhotra et al. (29) found that over half of the women (20/42) who had PBMV during pregnancy had a preterm delivery, with 50% of them having an iatrogenic birth as a result of pregnancy problems, compared to earlier studies that showed <10% preterm deliveries.

### 3.5 Long-term outcome of post-balloon mitral valvotomy

Ventricular volume changes after BMV were reported in a number of earlier investigations. Using an angiography and echocardiography, Mohan et al. (31) found no discernible change in left ventricular end-diastolic volume (LVEDV) or end-systolic volume (ESV) after BMV. Conversely, Goto et al. (32) used ventriculography to show an instantaneous rise in left-ventricular end-diastolic volume index (LVEDVI) following a successful BMV, and more recently, Sengupta et al. (25, 26) used echocardiography to reveal a significant increase in LVEDVI 72 h after BMV. According to the Fawzy et al. Study (1996) (23), there was a notable rise in LVEDVI just after BMV and another increase at the average 12-month follow-up. One year after BMV, Samaan et al. (3) demonstrated a late and significant improvement in left-ventricular ejection fraction (LVEF).

Numerous earlier investigations have reported increased afterload in MS patients. These patients’ decreased stroke volume may cause a compensatory peripheral systemic vasoconstriction, which would raise SVR. LV malfunction may be linked to abnormal preload. The development of intra-cardiac flow imaging tools has demonstrated that the pattern and quantity of diastolic filling are just as important to left ventricular systolic function. Maintaining a normal diastolic and systolic function requires that the flow within the heart chamber follow a complex sequence that is typified by the creation of spiral rings. The disruption of the typical filling pattern caused by mitral stenosis may be a contributing factor to the systolic dysfunction observed in MS patients. One of the variables linked to better LV performance after BMV may be an enhanced pattern of ventricular filling (31–34).

A prior pathological investigation that found ultra-structural pathological abnormalities in myocardial biopsies taken from MS patients provided the strongest evidence for the role of myocardial factor as a cause of left ventricular dysfunction in MS patients, aside from disrupted loading circumstances. Some people connected these pathological alterations to a past rheumatic myocardial process, whereas others connected them to persistent irregularities in ventricular filling (31–34).

## 4 Conclusion

The veno-arterial loop technique is a feasible and safe technique to facilitate mitral valve crossing for balloon mitral valvuloplasty in mitral stenosis patients. The technique is particularly beneficial in patients where valve crossing is not possible in the traditional way. This technique also gives more support for Inoue balloon entry across the LV compared to the over-the-wire technique.

For special population, based on data from observational studies, PBMV may be a safe and effective treatment for improving outcomes in MS women without significant subvalve disease. A successful PBMV significantly lowers mortality and helps tolerating hemodynamic alterations during pregnancy. Nonetheless, there is little literature on women with inadequate valve morphology who are not contraindicated, and more research is needed to determine whether PBMV is used during pregnancy in these cases. In the other hand, AF results in a decreased success rate for PBMV and inferior long-term and in-hospital outcomes.

## Data Availability

The original contributions presented in the study are included in the article/Supplementary Material, further inquiries can be directed to the corresponding author.
